# The improvement effect of working through the Silver Human Resources Center on pre-frailty among older people: a two-year follow-up study

**DOI:** 10.1186/s12877-023-03978-z

**Published:** 2023-05-03

**Authors:** Kumi Morishita-Suzuki, Momomi Nakamura-Uehara, Tomoaki Ishibashi

**Affiliations:** Dia Foundation for Research on Ageing Societies, VERDE VISTA Shinjuku-Gyoen, 3F, 1-34-5, Shinjuku, Tokyo, 160-0022 Japan

**Keywords:** Frailty, Pre-frailty, Silver Human Resources Center, Working in old age

## Abstract

**Background:**

Although the health benefits of working in old age are well known, no research has examined them among older people with pre-frailty. We examined the improvement effect of working through the Silver Human Resources Center (SHRC) on pre-frailty among older people in Japan.

**Methods:**

We carried out a two-year longitudinal survey from 2017 to 2019. Among 5,199 older people, the analysis included 531 participants who were judged to be of pre-frailty status at baseline and who completed both surveys. We utilized the records of participants’ work through the SHRC from 2017 to 2019. The evaluation of the frequency of working through the SHRC was categorized as “less-working” (i.e., less than a few times a month), “moderate-working” (i.e., one to two times a week), and “frequent-working” (i.e., more than three times a week). The transition of frailty status was classified as “improved” (from pre-frailty to robust) and “non-improved” (from pre-frailty to pre-frailty or frailty). Logistic regression was used to assess the influence of the frequency of working through the SHRC on the improvement of pre-frailty. The analysis model was adjusted for age, sex, working for financial reward, years of membership, community activities, and health status at baseline. Inverse-probability weighting was used to correct for survival bias in the follow-up period.

**Results:**

The improvement rate of pre-frailty during follow-up was 28.9% among the less-working, 40.2% in the moderate-working, and 36.9% in the frequent-working groups. The improvement rate in the less-working group was significantly lower than that in the other two groups (φ = -2.4). Multivariable logistic regression analysis showed that individuals in the moderate-working group had significantly higher odds of pre-frailty improvement than those in the less-working group (OR: 1.47, 95% CI: 1.14–1.90), and no significant differences were found between the frequent-working and less-working groups.

**Conclusions:**

We found that the participants engaged in moderate working through the SHRC significantly increased their rate of pre-frailty improvement, while frequent working showed no significant association. Therefore, in the future it is important to provide moderate work to older people with pre-frailty according to their health status.

**Supplementary Information:**

The online version contains supplementary material available at 10.1186/s12877-023-03978-z.

## Background

The health benefits of working in old age are well known. Some systematic reviews reported that working beyond the statutory retirement age exerts positive effects on health outcomes, including self-rated health, depression, and activities of daily living [1, 2].

Most previous studies, however, excluded older people with age-related dysfunction from the analysis sample. Thus, no research has investigated the health effects of working among older people with age-related dysfunction. This may be because older people with poor health have lower employment rates than healthy older people, making follow-up more difficult. Many older people decide to retire due to age-related functional decline [3]. Therefore, investigating the health benefits of working with older people with age-related functional decline will contribute to promoting working in old age.

Frailty is an age-related physiological syndrome that puts older people at higher risk for adverse health outcomes such as falls, institutionalization, hospitalization, and death [4–7]. Therefore, it is essential to prevent frailty in older people. As frailty is a reversible condition, intervention in early-stage “pre-frailty” is critical [8]. Many interventions for pre-frailty and frailty such as exercise [9, 10], nutritional support [11], cognitive training [12], and their combinations [12, 13] have been substantiated. In addition, a potential strategy for postponing frailty is the encouragement of social participation. However, the health effects of working on frailty status are unknown.

Japan has one of the most rapidly aging populations in the world. The prevalence of frailty, pre-frailty, and robust status was 8.7%, 40.8%, and 50.5% in 2012, respectively [14], indicating that pre-frailty is a common condition among older people in Japan. Despite this, Japanese older people have higher motivation to work than those in other developed countries [15]. The national survey in Japan has shown that 58.9% of people aged ≥ 60 years want to remain in paid work beyond the age of 70 years [16].

In Japan, one of the social resources for providing part-time work for older people is the Silver Human Resources Center (SHRC). Japanese older people aged ≥ 60 years can register with the SHRC without any upper age limit. There are 1,335 SHRC locations and 698,419 older people were registered as at 2021 [17]. The SHRC is the most popular option for older people in Japan who hope to work for their well-being [18] and not for financial reward. SHRC members are mainly engaged in simple light work such as cleaning (Additional File 1) [17, 19]. A previous study reported that working through the SHRC was associated with preventing the deterioration of higher-level functional capacity in daily living [20]. In recent years, the number of very old members has been increasing [17], and some members presented with physical and cognitive dysfunction [21]. However, no study has reported the health effects of working through the SHRC among members with frailty.

The present study examined the improvement effect of working on pre-frailty after two years among Japanese SHRC members. In addition, this study used inverse-probability weighting (IPW) to correct for survival bias in the follow-up period.

## Methods

### Study design and participants

This was a two-year longitudinal study conducted on older people who registered with an SHRC in an urban area in Saitama prefecture, Japan. This area had a population of approximately 1,330,000 in 2021 with 23.1% of the population being at least 65 years old in December 2021 [22]. At baseline in 2017, the study participants were all SHRC members aged ≥ 60 years. The exclusion criteria were as follows: (1) people who identified as robust or frailty, (2) people who received long-term care insurance, and (3) people with missing data.

**Fig. 1:**
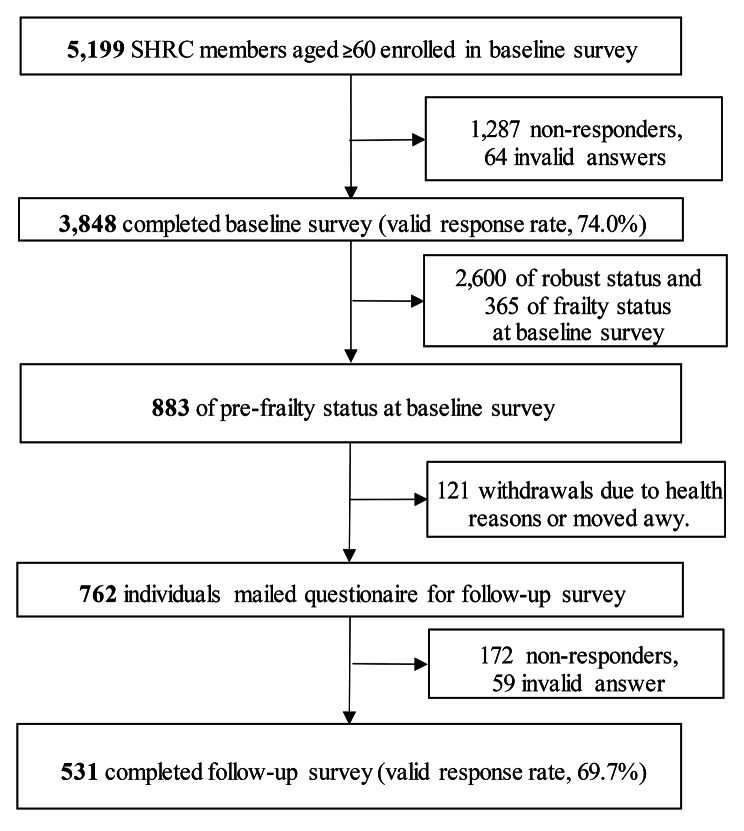
Flow of participants in the cohort study

Figure 1 shows a flowchart of this study’s participants. A total of 5,199 older people were enrolled in the baseline survey, which was carried out by mail in 2017 and 2019. These surveys were conducted prior to the first COVID-19 pandemic in Japan (March-May 2020) and had no impact on SHRC projects at the time of the study. In the baseline survey, participants were asked to complete a self-reported questionnaire. A total of 3,848 individuals completed the survey (valid response rate, 74.0%). We carried out a follow-up survey for 883 participants who had been identified as being in a pre-frailty state at baseline. In the follow-up period, 121 participants withdrew from the SHRC because of health reasons and having moved away from the study area. In the follow-up survey, a total of 762 individuals completed the survey. Of those, we included 531 participants with no missing survey items at follow-up for analysis (valid response rate 69.7%). The questionnaire was mailed together with a document describing the study purpose, which stated that cooperation was voluntary.

This study was conducted in accordance with the 1964 Declaration of Helsinki and its later amendments. The study was approved by the ethics committee of the Dia Foundation for Research on Ageing Societies (no. A29001). Informed consent was obtained from all individual participants in the study.

## Measurements

### Frailty assessment

Frailty was evaluated using the Kihon Checklist (KCL). The measurement is a self-reporting questionnaire consisting of 25 items, including five items for activities of daily living, five for physical function, two for nutritional status, three for oral function, two for house-boundedness, three for cognitive function, and five for depression. The sum of all indices’ questions ranged from 0 to 25, with a higher KCL score indicating a higher risk for requiring support or care. In this study, according to the frailty criteria [23], a KCL score of 0 to 3 was considered as robust, 4 to 7 as pre-frailty, and ≥ 8 as frailty. The transition of participants to frailty status was classified into two categories based on changes between 2017 and 2019: improved and non-improved transitions. We defined changes in pre-frailty according to the status at follow-up. The change was classified as “improved” (from pre-frailty to robust) and “non-improved” (from pre-frailty to pre-frailty or frailty).

### Frequency of working through the SHRC

We utilized the record data of working through the SHRC from 2017 to 2019. The evaluation of the frequency of working through the SHRC was categorized as “less-working” (i.e., less than a few times a month), “moderate-working” (i.e., one to two times a week), and “frequent-working” (i.e., more than three times a week). A previous study reported that working for about six hours a week prevented the deterioration of high-level functional capacity in daily living among SHRC members [20]. Since working through the SHRC involves working for about three to four hours per day [17], this study used twice a week as the cutoff to separate “moderate-working” from “frequent-working.”

### Covariates

As covariates, the following items were used: age, sex (male/female), working for financial reward (yes/no), years of membership, community activities, and health status at baseline. Age and years of membership were used as continuous variables. A work motive for financial reward is associated with reducing health benefits through working in old age [24]. Community activities were surveyed with the following question: “Do you participate in community activities other than the SHRC, such as senior clubs, income-generating jobs, volunteer activities, or neighborhood associations at least once a month?” Health status at baseline was assessed by six dimensions in the KCL: physical dysfunction, malnutrition, oral dysfunction, house-boundedness, cognitive dysfunction, and depression. These variables were examined in the baseline survey.

### Statistical analysis

The analysis was based on the data of 531 participants. The characteristics (age, sex, working for financial reward, years of membership, community activity, and health status at baseline) of the participants were compared with the frequency of working through the SHRC using the χ^2^ -test for categorical variables and Kruskal-Wallis test of variance for continuous variables. The improvement rate of pre-frailty was compared with the frequency of working through the SHRC using the χ^2^ -test and residual analysis.

Logistic regression was used to assess the influence of the frequency of working through the SHRC on the improvement of pre-frailty. The analysis model was adjusted for all of the covariates described above.

In this study, the follow-up rates were 60.1% (valid response n = 883 at baseline survey). To examine the possibility of dropout bias in follow-up, differences in attributes (age, sex, community activity, years of membership, and health status) between the follow-up and non-follow-up groups (n = 352) were analyzed by the χ^2^ test or Kruskal-Wallis test. When a variable with a difference of less than 10% probability of significance between these two groups was found, IPW was used to analyze the longitudinal data. Propensity scores for the IPW method were calculated by logistic regression and the trend score was calculated by logistic regression analysis [25]. All statistical analyses were performed with SPSS version 27.0 (IBM Japan, Ltd., Tokyo, Japan).

## Results

Table 1 shows the participants’ characteristics by frequency of working through the SHRC. There were significant differences in sex (p = 0.015) and community activities (p = 0.004) while there was no significant difference in the top five types of work content among the three groups (Additional File 2).

**Table 1:** Baseline characteristics of study participants

		Less-working (n = 162)					Moderate-working (n = 166)					Frequent-working (n = 203)				p-value
		n	(	%	)		n	(	%	)		n	(	%	)	
Age	Median (25 − 75%)	73.0	(	69.8–76.0	)		73.0	(	69.0-75.3	)		73.0	(	70.0–75.0	)	n.s
Sex	Male	107	(	66.0	)		117	(	70.5	)		161	(	79.3	)	**0.015**
Working for financial reward	Yes	16	(	9.9	)		13	(	7.8	)		18	(	8.9	)	n.s
Years of membership	Median (25 − 75%)	7.3	(	4.5–11.5	)		7.0	(	4.2–10.9	)		7.7	(	4.8–10.8	)	n.s
Community activities	Yes	105	(	64.8	)		91	(	54.8	)		96	(	47.3	)	**0.004**
Health status at baseline																
	Physical dysfunction	15	(	9.3	)		10	(	6.0	)		20	(	9.9	)	n.s
	Malnutrition	3	(	1.9	)		3	(	1.8	)		1	(	0.5	)	n.s
	Oral dysfunction	36	(	22.2	)		33	(	19.9	)		51	(	25.1	)	n.s
	Cognitive dysfunction	53	(	32.7	)		55	(	33.1	)		59	(	29.1	)	n.s
	House-boundness	1	(	0.6	)		1	(	0.6	)		2	(	1.0	)	n.s
	Depression	63	(	38.9	)		58	(	34.9	)		61	(	30.0	)	n.s

Total n = 531

Less-working, working less than a few times a month; Moderate-working, working one to two times a week; Frequent-working, working more than three times a week

Differences between groups in baseline characteristics were evaluated using the χ^2^ -test for categorical variables and the Kruskal-Wallis test of variance for continuous variables; Bold: p < 0.05

Table 2 shows the improvement rate of pre-frailty during follow-up. Among participants with pre-frailty at baseline, 28.9% were in the less-working, 40.2% were in the moderate-working, and 36.9% were in the frequent-working groups. The results of χ^2^ revealed significant differences in frequency of work (χ^2^(2) = 6.384, p = 0.041). Residual analysis revealed that the improvement rate in the less-working group was significantly lower than in the other two groups (φ = -2.4). There were no significant differences between the moderate-working and frequent-working groups.

**Table 2:** The improvement rate of pre-frailty after two years by frequency of working through the SHRC

	Less-working (n = 162)					Moderate-working (n = 166)					Frequent-working (n = 203)					p-value
	%	(	z	)		%	(	z	)		%	(	z	)		
Improved	28.4	(	**-2.4**	)		41.6	(	1.9	)		36.9	(	0.4	)		**0.041**

Total n = 531

Improved, changes from pre-frailty to robust.

Difference between groups in rate of improvement was evaluated using the χ2 -test and residual analysis.

Table 3 shows the results of logistic regression analysis, which examined the influence of the frequency of working through the SHRC on pre-frailty. Individuals in the moderate-working group were more likely to show improvement than those in the less-working group (Odds ratio: 1.47, 95% Confidence interval: 1.14–1.90, p = 0.003). However, individuals in the frequent-working group were not significantly different from those in the less-working group (Odds ratio: 1.12, 95% Confidence interval: 0.87–1.45). These analyses used IPW.

**Table 3:** Impact of working through the SHRC on the improvement of pre-frailty after two years

		OR		95%CI				p-value
Frequency of working	(ref. Less-working)							
	Moderate-working	1.47	(	1.14	-	1.90	)	**0.003**
	Frequent-working	1.12	(	0.87	-	1.45	)	n.s.
Age	(year)	0.98	(	0.95	-	1.01	)	n.s.
Sex	Male (ref. Female)	0.83	(	0.66	-	1.05	)	n.s.
Working for financial reward	Yes (ref. No)	0.79	(	0.55	-	1.14	)	n.s.
Years of membership	(year)	1.00	(	0.97	-	1.03	)	n.s.
Community activities	Yes (ref. No)	1.11	(	0.91	-	1.36	)	n.s.
Health status at baseline								
	Physical dysfunction	0.68	(	0.47	-	0.98	)	**0.036**
	Malnutrition	1.20	(	0.43	-	3.33	)	n.s.
	Oral dysfunction	0.72	(	0.56	-	0.92	)	**0.009**
	Cognitive dysfunction	0.29	(	0.24	-	0.36	)	**< 0.001**
	House-boundness	3.97	(	0.84	-	18.65	)	n.s.
	Depression	0.54	(	0.42	-	0.68	)	**< 0.001**

Total n = 531. Logistic regression analysis; Bold: p < 0.05

Odds ratio (OR)

>1 indicates a likely increase in improving pre-frailty; 95% CI, 95% confidence interval

This model adjusted for missing data using Inverse-probability weighting (IPW)

## Discussion

The present study examined the association between working through the SHRC and pre-frailty over two years among Japanese older people. Our findings suggest that moderate working through the SHRC was associated with an increased probability of improving pre-frailty among older people.

Among this study’s participants at baseline, the prevalence of pre-frailty was 22.9%, which is lower than in a previous study using a nationally representative sample in Japan (40.8%) [14]. This may be because the older people who register with SHRCs are likely to be in good health and to have high motivation to participate in society. Another possible reason is that the target area was in eastern Japan, and the prevalence of frailty there tends to be lower than in western Japan [14].

In addition, 72.0% of individuals with pre-frailty worked at least once a week for two years. A total of 30.9% of Japanese older people aged 65–69 years who wanted to work but could not find work cited their health conditions as the reason [3]. In the future, it is hoped that by elucidating the mechanisms that enable SHRC members to work even if they are of pre-frailty status, the social participation of older people of pre-frailty status can be promoted with their willingness.

The results of this study showed that the improvement rate from pre-frailty to robust status was 35.8%. A meta-analysis including a Japanese cohort study of older people reported that the rate was 23.1% [8]. These estimates vary depending on the population studied and the definition of frailty. While these studies were based on physical markers [4], we used a broader multidimensional approach, namely the KCL. A systematic review revealed that studies using certain scales based on physical function reported a lower frailty prevalence than those using a broad definition (i.e., a multidimensional concept such as comprehensive geriatric assessment) of frailty among community-dwelling older people [26]. In addition, most of the items in the KCL are questions about performance, not capacity [23]. Therefore, moderate working through the SHRC may have served as a lifestyle intervention and may have increased the implementation of performance related to activities of daily living.

In the present study, we carried out logistic regression analyses to examine the association between working through the SHRC and pre-frailty over two years among Japanese older people. Our findings suggested that moderate working (i.e., one to two times a week) was associated with an increased probability of improving pre-frailty compared with less working, and that frequent working (i.e., ≥ three times a week) was not significantly different from less working. One previous study suggested a U-shaped relationship between total hours of unpaid and paid work per day and health outcomes such as cognitive impairment, activities of daily living, and death, with about eight hours of activity per day lowering health risks the most after three years among Japanese older people aged ≥ 70 years [27]. Another previous study suggested that moderate participation (i.e., less than a few times a month) in volunteer groups rather than frequent-working participation (i.e., at least once a week) may produce beneficial effects in the prevention of instrumental activities of daily living decline after two years among older Japanese women [28]. Although the results of the present study and these findings are not very comparable, they indicate that moderate participation in a productive activity may also be effective in improving the health status of older people with pre-frailty. In addition, since frequent working is not significantly related to pre-frailty improvement, it will be important for the SHRC office to provide moderate-working opportunities for members based on their health status.

The working hours per day at the SHRC are about three to four hours [17]. A previous study reported that working through the SHRC involves approximately 30 min of moderate to high-intensity physical activity of three metabolic equivalents (METs) or more [21]. The standard for physical activity in Japanese is “people aged ≥ 65 years should engage in 40 minutes of physical activity daily other than lying down or sitting” [29]. Therefore, working through the SHRC even a few times a week will contribute significantly to achieving the desired physical activity standard.

In addition, the SHRC’s work is team-based, so there is a lot of interaction with colleagues. Since there are no hierarchical relationships and the relationships are equal, there are fewer relationship stressors that can negatively impact mental health [30]. The systematic reviews showed that multi-domain intervention (e.g., physical exercise plus cognitive training) in older people with pre-frailty or frailty was more effective in group sessions than in one-on-one sessions [31]. Thus, the physical activity and interpersonal interaction associated with working through the SHRC may have been effective in improving pre-frailty. However, the mechanism cannot be clarified in this study, and future qualitative studies are warranted.

Individuals with a risk of physical dysfunction, oral dysfunction, cognitive dysfunction, and depression were associated with decreased probability of improving pre-frailty compared with those with no risk. A previous study reported that individuals with cognitive and physical dysfunction might experience fatigue at work [21]. In addition, other previous studies reported that these dysfunctions were significantly associated with new certifications in long-term care [32]. In the future, programs to improve these dysfunctions should be implemented along with the provision of jobs, and the effectiveness of such programs should be verified.

A major strength of the present study was the enrollment of older workers with pre-frailty. Previously, most research investigating health outcomes only involved healthy older workers, since older workers with poor health are more likely to drop out. Even among the very old, when the prevalence of frailty is high [14], many older people wish to continue working [16]; thus, the present findings help us to understand the relationship between working in older age and health.

The present study had several limitations that must be mentioned. First, in all cases, health status was self-reported. We used a measure that has reportedly acceptable measurement properties, but participants could have overestimated their health status. Further studies should use objective health outcomes to evaluate the impact of working on health benefits. Second, this study was limited to a single urban area with higher income in Japan [33]. Further research should be conducted in rural municipalities with the opposite characteristics. Third, workload was not measured. Although the survey confirmed that there was no difference in work content by the frequency of working, individuals may have different workloads for the same work content. Fourth, we cannot deny the possibility of residual confounding. For example, we lacked measures of socioeconomic status (i.e., education and income) and lifestyle habits (i.e., smoking and physical activity). This indicates that there may be subgroups with varying effects of working on pre-frailty improvement, which we were unable to account for in our analysis. Thus, future studies exploring the interaction between working status and individual characteristics, including those aforementioned, are needed to further elucidate these potential heterogeneous effects. Finally, the impact of working on health among older people with pre-frailty may have not only positive effects, but also negative effects such as accidents that were not considered in this study. Future research on risk management among older workers with pre-frailty may be necessary.

## Conclusions

Moderate working (i.e., one to two times a week) through the SHRC significantly increased the rate of pre-frailty improvement, while frequent working (i.e., more than three times a week) showed no significant association. Finally, the COVID-19 pandemic limited social participation, including working, among older people worldwide. Currently, the provision of work in SHRC is recovering in Japan and is expected to become an important recipient of social participation among older people after the pandemic. In the future, as the results of this study indicate, it is desirable to provide moderate work opportunities for older people according to their health status.

## Electronic supplementary material

Below is the link to the electronic supplementary material.

Supplementary Material 1

Supplementary Material 2

Supplementary Material 3

## Data Availability

The datasets generated and/or analyzed during the current study are not publicly available due to them containing information that could compromise research participant privacy, but are available from the corresponding author on reasonable request.

## Abbreviations

IPW: inverse-probability weighting
KCL: Kihon Checklist
METs: metabolic equivalents
SHRC: Silver Human Resources Center

## References

[1] Van der Noordt M, Ijzelenberg H, Droomers M, Proper KI (2014). Health effects of employment: a systematic review of prospective studies. Occup Environ Med.

[2] Baxter S, Blank L, Cantrell A, Goyder E (2021). Is working in later life good for your health? A systematic review of health outcomes resulting from extended working lives. BMC Public Health.

[3] The Japan Institute for Labour Policy and Training. : Survey on the employment and employment status of older persons. https://www.jil.go.jp/institute/research/2010/075.html (2010). Accessed 24 Mar 2022.

[4] Fried LP, Tangen CM, Walston J, Newman AB, Hirsch C, Gottdiener J (2001). Frailty in older adults: evidence for a phenotype. J Gerontol A Biol Sci Med Sci.

[5] van Abellan GA, Rolland Y, Bergman H, Morley JE, Kritchevsky SB, Vellas B (2008). The I.A.N.A. Task Force on frailty assessment of older people in clinical practice. J Nutr Health Aging.

[6] Bergman H, Ferrucci L, Guralnik J, Hogan DB, Hummel S, Karunananthan S (2007). Frailty: an emerging research and clinical paradigm—issues and controversies. J Gerontol A Biol Sci Med Sci.

[7] Tom SE, Adachi JD, Anderson FA, Boonen S, Chapurlat RD, Compston JE (2013). Frailty and fracture, disability, and falls: a multiple country study from the global longitudinal study of osteoporosis in women. J Am Geriatr Soc.

[8] Kojima G, Taniguchi Y, Iliffe S, Jivraj S, Walters K (2019). Transitions between frailty states among community-dwelling older people: a systematic review and meta-analysis. Ageing Res Rev.

[9] de Vries NM, van Ravensberg CD, Hobbelen JSM, Olde Rikkert MGM, Staal JB, Nijhuis-van der Sanden MWG (2012). Effects of physical exercise therapy on mobility, physical functioning, physical activity and quality of life in community-dwelling older adults with impaired mobility, physical disability and/or multi-morbidity: a meta-analysis. Ageing Res Rev.

[10] Clegg AP, Barber SE, Young JB, Forster A, Iliffe SJ (2012). Do home-based exercise interventions improve outcomes for frail older people? Findings from a systematic review. Rev Clin Gerontol.

[11] Kim CO, Lee KR (2013). Preventive effect of protein-energy supplementation on the functional decline of frail older adults with low socioeconomic status: a community-based randomized controlled study. J Gerontol A Biol Sci Med Sci.

[12] Ng TP, Feng L, Nyunt MSZ, Feng L, Niti M, Tan BY (2015). Nutritional, physical, cognitive, and combination interventions and frailty reversal among older adults: a randomized controlled trial. Am J Med.

[13] Luger E, Dorner TE, Haider S, Kapan A, Lackinger C, Schindler K (2016). Effects of a home-based and volunteer-administered physical training, nutritional, and social support program on malnutrition and frailty in older persons: a randomized controlled trial. J Am Med Dir Assoc.

[14] Murayama H, Kobayashi E, Okamoto S, Fukaya T, Ishizaki T, Liang J (2020). National prevalence of frailty in the older japanese population: findings from a nationally representative survey. Arch Gerontol Geriatr.

[15] Cabinet Office. The 9th International Comparative Survey on the Lives and Attitudes of the Elderly. 2021. https://www8.cao.go.jp/kourei/ishiki/r02/zentai/pdf/2_5.pdf. Accessed 2 Feb 2022.

[16] Ministry of Health Labour and Welfare. Annual report on the ageing society. 2020. https://www8.cao.go.jp/kourei/english/annualreport/2020/pdf/2020.pdf. Accessed 9 Nov 2021.

[17] National Silver Human Resources Center Association (2020). Annual statistical report. NRI Soc Inf Syst Serv Tokyo.

[18] Weiss RS, Bass SA, Heimovitz HK, Oka M (2005). Japan’s silver human resource centers and participant well-being. J Cross Cult Gerontol.

[19] Ishibashi T, Morishita K, Nakamura M. Changes in the work style of members of Silver Human Resources Centers over 10 years. 2020;42:209–16. 10.34393/rousha.42.3_209.

[20] Nakamura M, Osada H, Sugisawa S (2016). The effect of working in a silver human resource center in a Tokyo suburb on cognitive higher-level functional capacity in daily living. J Gerontol Res.

[21] Morishita K, Watanabe S, Osada H (2020). Influence of motor function and cognitive function on self-perceived fatigue: examination of outdoor work by older people registered in Silver Human Resources Center. J Appl Gerontol.

[22] Saitama City. : Population and Households in Saitama City. https://www.city.saitama.jp/006/013/005/001/p077762.html (2021). Accessed 9 Nov 2021.

[23] Satake S, Senda K, Hong YJ, Miura H, Endo H, Sakurai T (2016). Validity of the Kihon Checklist for assessing frailty status. Geriatr Gerontol Int.

[24] Nemoto Y, Takahashi T, Nonaka K, Hasebe M, Koike T, Minami U (2020). Working for only financial reasons attenuates the health effects of working beyond retirement age: a 2-year longitudinal study. Geriatr Gerontol Int.

[25] Seaman SR, White IR (2013). Review of inverse probability weighting for dealing with missing data. Stat Methods Med Res.

[26] Collard RM, Boter H, Schoevers RA, Oude Voshaar RC (2012). Prevalence of frailty in community-dwelling older persons: a systematic review. J Am Geriatr Soc.

[27] Shibata H, Sugihara Y, Sugisawa H (2012). Determinants and effects on the well-being of productive activities in late-middle-aged and aged Japanese: longitudinal analysis of two representative panels. J Appl Gerontol.

[28] Tomioka K, Kurumatani N, Saeki K (2018). The differential effects of type and frequency of social participation on IADL declines of older people. PLoS ONE.

[29] Ministry of Health Labour and Welfare. : Physical activity standards for Health Promotion. https://www.mhlw.go.jp/stf/houdou/2r9852000002xple.html (2013). Accessed 11 Apr 2022.

[30] National Silver Human Resources Center Association. Research Report on the effectiveness of Long-Term Care Prevention among Silver Human Resources Center Members. National Silver Human Resources Center Association (Tokyo; 2022. pp. 12–22.

[31] Apóstolo J, Cooke R, Bobrowicz-Campos E, Silvina S, Maura M, Antonio C (2018). Effectiveness of interventions to prevent pre-frailty and frailty progression in older adults: a systematic review. JBI Database Syst Rev Implement Rep.

[32] Morita Y, Arai T, Fujita H, Watanabe S (2021). Two-year changes in the kihon checklist and initiations of long-term care in community-dwelling elderly. Rigakuryoho Kagaku.

[33] Cabinet Office. : Population and economic data by the municipality. https://www5.cao.go.jp/keizai-shimon/kaigi/special/future/keizai-jinkou_data.html (2014). Accessed 2 Feb 2022.

