# Supplementary material for: The improvement effect of working through the Silver Human Resources Center on pre-frailty among older people: a two-year follow-up study
Source: BMC Geriatr. 2023 May 3;23:265. doi: 10.1186/s12877-023-03978-z (PMC10155134; doi:10.1186/s12877-023-03978-z)
Supplement: Supplementary file 1 — Supplementary Material 1 [file 12877_2023_3978_MOESM1_ESM.docx]

**Supplementary Table 1. The main work content of the Silver Human Resources Center in 2021**

| Category | Work content | % |
| --- | --- | --- |
| Transportation, cleaning, packaging | Light work inside or outside (i.e., cleaning), grass trimming, shopping cart organization, etc. | 46.5 |
| Agriculture, forestry, and fisheries | Tree pruning, agricultural support, landscaping work, etc. | 34.5 |
| Services | Building management, advertising distribution, welfare work and help with domestic tasks, childcare support, public facility management, school crossing guard, etc. | 9.3 |
| Production processing | Mending clothes, knife sharpening, chip and compost production, picture mounting, etc. | 4.8 |
| Office work | General office work, accounting, etc. | 1.9 |
| Construction and mining | Furniture repair, interior decoration work, etc. | 1.8 |
| Specialized and technical | Instructor, translation, interpretation, etc. | 0.6 |
| Transportation and machinery operation | Vehicle driving, etc. | 0.3 |
| Sales | Store workers, salespeople, etc. | 0.2 |
