# Supplementary material for: The improvement effect of working through the Silver Human Resources Center on pre-frailty among older people: a two-year follow-up study
Source: BMC Geriatr. 2023 May 3;23:265. doi: 10.1186/s12877-023-03978-z (PMC10155134; doi:10.1186/s12877-023-03978-z)
Supplement: Supplementary file 2 — Supplementary Material 2 [file 12877_2023_3978_MOESM2_ESM.docx]

| Supplementary Table.2 Comparison of baseline characteristics of analytes and dropout groups | | | | | | | | | | | |
| --- | --- | --- | --- | --- | --- | --- | --- | --- | --- | --- | --- |
|  |  | Analytes  (n=531) | | |  |  | Dropout  (n=352) | | |  | p-value |
|  |  | n | ( | % | ) |  | n | ( | % | ) |  |
| Age | Median (25% - 75%) | 73.0 | ( | 69.0-76.0 | ) |  | 73.0 | ( | 69.0-77.0 | ) | n.s. |
| Sex | Male | 385 | ( | 72.5 | ) |  | 238 | ( | 67.6 | ) | n.s. |
| Working for financial rewards | yes | 47 | ( | 8.9 | ) |  | 38 | ( | 10.8 | ) | n.s. |
| Year of enrollment | Median (25% - 75%) | 7.3 | ( | 4.6-11.1 | ) |  | 7.3 | ( | 3.9-11.5 | ) | n.s. |
| Community activities | yes | 292 | ( | 55.0 | ) |  | 174 | ( | 49.4 | ) | n.s. |
| Health status at baseline |  |  |  |  |  |  |  |  |  |  |  |
|  | Physical dysfunction | 45 | ( | 8.5 | ) |  | 38 | ( | 89.2 | ) | n.s. |
|  | Mulnutrition | 7 | ( | 1.3 | ) |  | 3 | ( | 0.9 | ) | n.s. |
|  | Oral dysfunction | 120 | ( | 22.6 | ) |  | 73 | ( | 20.7 | ) | n.s. |
|  | Cognitive dysfunction | 167 | ( | 31.5 | ) |  | 23 | ( | 6.5 | ) | **<0.001** |
|  | Houseboundness | 4 | ( | 0.8 | ) |  | 4 | ( | 1.1 | ) | n.s. |
|  | Depressive mood | 182 | ( | 34.3 | ) |  | 146 | ( | 41.5 | ) | **0.030** |
| Differences between groups in baseline characteristics were evaluated using the χ^2^ -test for categorical variables and Kruskal-Wallis test of variance for continuous variables. | | | | | | | | | | | |
