# Supplementary material for: The improvement effect of working through the Silver Human Resources Center on pre-frailty among older people: a two-year follow-up study
Source: BMC Geriatr. 2023 May 3;23:265. doi: 10.1186/s12877-023-03978-z (PMC10155134; doi:10.1186/s12877-023-03978-z)
Supplement: Supplementary file 3 — Supplementary Material 3 [file 12877_2023_3978_MOESM3_ESM.docx]

**Supplementary Table 3. Top five types of work content**

|  |  | Moderate-working  (n=162) | Frequent-working  (n=203) | p-value |
| --- | --- | --- | --- | --- |
|  |  | % | % |  |
| 1 | Light work outdoors (i.e., cleaning a park) | 42.2 | 45.3 | n.s |
| 2 | Light work indoors (i.e., cleaning in a building) | 33.7 | 36.5 | n.s |
| 3 | Building management | 29.5 | 28.1 | n.s |
| 4 | Welfare work and help with domestic tasks | 17.5 | 10.8 | n.s |
| 5 | General office work | 13.3 | 10.8 | n.s |
| The difference between groups in the top five work content was evaluated using the χ^2^ -test. | | | | |
